# Beef- and Pork-Based Dishes from Catering Services: Composition and In Vitro Digestion Effects on Digestibility and Lipid Oxidation

**DOI:** 10.3390/foods14050789

**Published:** 2025-02-25

**Authors:** Itziar Ariz-Hernandez, Patrick Schulz, Roncesvalles Garayoa, Diana Ansorena, Iciar Astiasaran

**Affiliations:** 1 Department of Nutrition, Food Science and Physiology, Faculty of Pharmacy and Nutrition, University of Navarra, Irunlarrea 1, 31008 Pamplona, Spain; iarizh@unav.es (I.A.-H.); pschulz@alumni.unav.es (P.S.); rgarayoa@unav.es (R.G.); iastiasa@unav.es (I.A.); 2 Center for Nutrition Research, Faculty of Pharmacy and Nutrition, University of Navarra, Irunlarrea 1, 31008 Pamplona, Spain; 3 IdiSNA, Navarra Institute for Health Research, 31008 Pamplona, Spain

**Keywords:** meat, INFOGEST, cooking method, oxidative status

## Abstract

Twelve meat-based dishes (beef/pork) prepared using different cooking methods and ingredients were collected from two catering services. Their nutritional composition and lipid oxidation status was analyzed. Subsequently, the samples underwent an in vitro digestion process to evaluate their digestibility and the effect of digestion on lipid oxidation. The protein content of the dishes ranged from 17% to 34%, with no clear influence from the type of meat or cooking method. Lipid content showed considerable variability (2.5–15.1%), with all dishes exhibiting a high omega-6/omega-3 ratio. In vitro dry matter digestibility ranged from 58% to 86%, protein digestibility from 77% to 93%, and lipid digestibility from 7.3% to 46%. Among all dishes, “roasted pork loin” showed the highest digestibility values. Regarding lipid oxidation, grilled samples exhibited the lowest levels before digestion (less than 0.85 ppm MDA), whereas most of the roasted dishes exceeded 4 ppm MDA. After digestion, all samples—except “stewed veal—a”—suffered an increase in oxidation. Stewed dishes had the smallest increase (less than 60%) and “roasted pork meatballs” exhibited the highest increase (more than 600%). This study enhances the knowledge of the nutritional value of meat-based dishes and the impact of the digestion process.

## 1. Introduction

Meat is widely recognized and valued around the world, not only for its richness in essential nutrients, but also for its versatility in cooking and its ability to adapt to a wide variety of culinary traditions and cultures. Red meat, including beef, lamb, goat or pork, is the most preferred meat, with a production of 206 million tons/annually [1]. This is a good source of high-quality protein, providing abundant amounts of essential amino acids [2,3,4], essential fatty acids, vitamin B, iron and zinc.

Although meat can be consumed raw, it is most commonly eaten cooked. Cooking enhances food safety by preventing the growth of microorganisms, inactivating enzymes, and extending its shelf life. Moreover, cooking develops a different odor, taste, color, and texture of meat properties due to the physico-chemical changes induced by the heat treatment, giving rise to a broad variety of dishes. There are several ways to cook it, ranging from traditional techniques (roasting, frying, grilling, stewing) to modern methods (microwaving, sous vide cooking, ohmic cooking). These techniques can positively or negatively affect protein digestibility, as well as the bioavailability of other nutrients. At a moderate temperature (70 °C), cooking causes protein denaturalization, increasing the enzymatic cleave sites, and thus improving the digestibility [5]. However, if meats are overcooked at temperatures above 100 °C, proteins suffer aggregation due to oxidation, impairing the enzymatic action and decreasing the amino acid availability [6].

Lipid compounds positively influence meat properties such as aroma, palatability, and texture, but raise health concerns due to the potential excess of cholesterol and saturated fatty acids. During cooking, the high temperature, and the presence of oxygen, light, and metal ions favor the formation of free radicals, inducing the oxidation of fatty acids. The intensity of these reactions depends on various factors, including the type of meat, time/temperature combinations, and the presence of pro-oxidant or antioxidant compounds [7]. Lipid oxidation is the main cause of rancidity, undesirable odors, texture modification, losses of essential fatty acids and deterioration of meats. It is also responsible for the formation of toxic compounds related to cardiovascular and neurodegenerative diseases, as well as mutagenic and carcinogenic disorders [8]. Malondialdehyde is a well-established lipid oxidation marker, formed as a secondary product from the peroxidation of omega-6 and omega-3 polyunsaturated fatty acids. It is known that it forms adducts with DNA and proteins, and alters the lipid functions raising health concerns [9].

In vitro digestion models, although being an approximation tool to study the digestion process occurring in the human organism, are widely used because of their easy applicability [10] and their usefulness in predicting the outcomes of in vivo digestion [11]. There is a lot of evidence about the increments of oxidation during the in vitro digestion process due to the gut prooxidant conditions, such as added oxygen or the low pH during gastric digestion [12,13,14]. Van Hecke et al. [15], in a review over the oxidation process taking place during the in vitro digestion of meat, pointed out that oxidative stress resulting from its consumption may mediate the onset and progression of a wide range of diseases.

The aim of this work was to provide information about the composition of various meat-based dishes supplied by catering services and the effect of the in vitro digestion process on their digestibility and oxidation status in order to conclude their real nutritional value and potential health implications.

## 2. Materials and Methods

Twelve cooked meat-based dishes (six with beef and six with pork) were provided by two different catering services from Navarra (Spain). The raw meats used for their preparation were subjected to different cooking treatments (grilling, stewing, and roasting), of which the detailed conditions are described in Table 1. The samples were collected gradually, and once received in the laboratory, they were homogenized and stored under vacuum at −20 °C until analysis.

### 2.1. General Composition

The ash, moisture, and protein contents of all samples were analyzed using the official methods 920.153, 950.46, and 928.08 [16,17,18]. The Soxhlet extractor B-811 Büchi Extraction System (BÜCHI Labortechnik AG, Flawil, Switzerland) was used to quantify the fat content [19]. These determinations were carried out in triplicate per each type of dish.

The carbohydrate content was then calculated by difference (100% minus the percentage of moisture, protein and ash) and the energy value was calculated using the following conversion factors: 4 kcal/g of protein or carbohydrate, and 9 kcal/g of fat. Results are shown in Table 2.

### 2.2. Fatty Acid Profile

For the determination of the fatty acid profile of the 12 cooked samples, a fat extraction was firstly performed using the Folch et al. [20] method. Then, the fat was derivatized into fatty acid methyl esters (FAMEs) using the official method [21]. Lastly, the analysis of the lipid profile was carried out in a gas chromatography with a flame ionization detector (FID) and a capillary column SP-2560 (100 m × 0.25 mm × 0.2 µm) on a Perkin Elmer Clarus 500 (Perkin Elmer, Shelton, CT, USA). The injector was set at 250 °C and the detector at 260 °C. The first 10 min, the oven was at 175 °C and then, the temperature increased firstly to 200 °C with a ratio of 10 °C/min and then to 220 °C with a rate of 4 °C/min, maintained for 15 min. Hydrogen with a pressure of 30 psi and split ratio of 120 mL/min was used as the carrier gas. As an internal standard, heptadecanoic acid methyl ester was used. The conversion of total fatty acid percentages to total fat was calculated in each dish taking into account the sum of all detected fatty acids (reached between 0.77 and 0.84 g fatty acids/g fat). The final results (Table 3) were expressed as g of fatty acids/100 g of dish, considering the fat amount of each dish. Additionally, the results expressed as g of fatty acid/100 g of total fatty acids are given in the Supplementary Materials. Analysis was performed in duplicate per each type of dish.

### 2.3. In Vitro Digestion

The 12 cooked samples were submitted to an in vitro digestion process following the INFOGEST method [11]. The protocol consists of three steps: oral, gastric, and intestinal. For the oral phase, 5 g of cooked samples were transferred to a flask and mixed with 4 mL simulated salivary fluid, 0.475 mL of water, 25 µL of 0.3 M CaCl_2_(H_2_O)_2_, and 0.5 mL of α-amylase (equivalent to 75 U/mL final digestion volume). The mixture was stirred and incubated at 37 °C for 2 min in a water bath. For the gastric phase, 8 mL of simulated gastric fluid, 5 µL of 0.3 M CaCl_2_(H_2_O)_2_, 0.5 mL of pepsin (equivalent 2000 U/mL final digestion volume), and 0.5 mL of lipase (equivalent 60 U/mL final digestion volume) were added, the pH was adjusted to 3 with HCL, and water was added to reach 20 mL. The mixture was then incubated at 37 °C for 2 h. For the intestinal step, 3.5 mL of simulated intestinal fluid, 2.5 mL bile salt (10 nM), 40 µL of 0.3 M CaCl_2_(H_2_O)_2_, 5 mL of pancreatin from porcine pancreas (equivalent 100 U/mL of trypsin), and 5 mL of lipase (equivalent 2000 U/mL final digestion volume) were added, the pH was adjusted to 7 using NaOH and water was added to reach 40 mL. The bioaccessible fraction (micellar phase) and the residual fraction (pellet) were separated by centrifugation at 2000 rcf 50 min (A-4-62 Rotor, Eppendorf centrifuge 5810R Eppendorf, Barcelona, Spain). Both fractions were then stored at −20 °C until analysis. For each sample, three digestions were carried out. A blank was also carried out using water to replace the sample.

### 2.4. Digestibility Parameters

The residual fraction or pellet of each digested sample and the blank were dried in an oven firstly at 80 °C for 6 h and then at 45 °C until constant weight. All sample weights were blank-corrected.

Equation (1) was used to calculate the in vitro dry matter digestibility (IVDMD), according to Khemiri et al. [22]:

(1) $$\mathrm{IVDMD}\;(\%)=\frac{DWS\;(g)-DWP\;(g)}{DWS\;(g)}\times 100$$

where DWS refers to the dry weight of the sample submitted to digestion and DWP refers to the dry weight of the pellet.

The Kjeldahl method [18] was used to analyze the protein content of dried pellet samples and the amount of protein was blank-corrected. Then, in vitro protein digestibility (IVPD) was calculated using Equation (2), following the method described previously [22]:

(2) $$\mathrm{IVPD}\;(\%)=\frac{PDS\;(g)-PDP\;(g)}{PDS\;(g)}\times 100$$

where PDS refers to the protein content of the dry sample submitted to digestion, and PDP refers to the protein content of the dry pellet.

For the in vitro lipid digestibility (IVLD), 4 g of the micellar phase was taken and the fat content was extracted using chloroform:methanol (2:1 *v*/*v*). Then, a titration with NaOH 0.01 M was performed to calculate the free fatty acid amount, using ethanol: diethyl ether (1:1 *v*/*v*) to dissolve the fat and phenolphthalein as an indicator. IVLD was calculated using Equation (3) [23]:

(3) $$\mathrm{IVLD}\;(g\;\mathrm{FFA}/100\;g\;\mathrm{fat})=\frac{\mathrm{Free}\mathrm{fatty}\mathrm{acids}\mathrm{amount}\mathrm{in}\mathrm{digested}\mathrm{micellar}\mathrm{phase}\;(g)}{\mathrm{Fat}\mathrm{amount}\mathrm{in}\mathrm{sample}\mathrm{submitted}\mathrm{to}\mathrm{digestion}\;(g)}\times 100$$

### 2.5. Lipid Oxidation

The thiobarbituric acid reactive substances (TBARSs) method was used as a lipid oxidation indicator in cooked samples before and after the in vitro digestion process (in the micellar fraction), following the method described by Sobral et al. [24]. Briefly, 150 mg of the cooked samples or 400 µL of digested micellar fraction were transferred to a microtube and completed to 1 mL with 7.5% TCA. Then, to avoid oxidation during the analysis, 40 µL of 2,6-di-tert-butyl-4-methylphenol (BHT) was added. Microtubes were then vortexed and centrifugated at 1680 rcf for 5 min (rotor F-45-12-11, Eppendorf^®^ Centrifuge MiniSpin G, Eppendorf, Barcelona, Spain). The resulted supernatant was transferred to another microtube. Then, 250 µL of 7.5% TCA was added again to the pellet for a second precipitation and then, it was again mixed in vortex and centrifugated at 3780 rcf 5 min. The supernatant of the second centrifugation was added to the previous supernatant. Then, 500 µL of the supernatant was transferred to a new microtube and mixed with 500 µL of 40 mM thiobarbituric acid (TBA) reagent. The mixture was vortexed and heated at 90 °C for 45 min. Then, the samples were cooled in an ice bath and 200 µL was transferred to a microplate to read the absorbance at 532 nm (FLUOStar Optima, Microplate Reader, BMG, Ortenberg, Alemania). As a calibration standard, 1,1,3,3-tetraethoxypropane (TEP) was used. The result was expressed as milligrams of malondialdehyde per kilograms of sample. All analyses were performed in triplicate.

### 2.6. Statistical Analysis

The tables depict the mean and standard deviations of all data obtained for the pseudo-replicates of each dish. A one-way analysis of variance (ANOVA) and the post hoc Tukey test were applied to evaluate statistical significance (*p* < 0.05) among dishes for each parameter. In the case of lipid oxidation, the Student *t*-test was applied to evaluate statistical significance (*p* < 0.05) between samples before and after digestion. The software used for all the analysis was the STATA 15 program (Stata Corp LLC, College Station, TX, USA).

## 3. Results and Discussion

Twelve dishes based on beef and pork were studied (six elaborated with beef and another six elaborated with pork). Table 1 shows the information about the heating treatments (cooking method, equipment, temperature, and time) and the ingredients used for the 12 meat dishes. Three different heat treatments were applied: grilling, stewing, and roasting. Olive oil was used in all the dishes, and in one of them, sunflower oil was also used. In 10 dishes, the recipes included vegetable ingredients (onion, pepper, carrot, garlic, pea, tomato, apple, parsley, leek, and/or bay leaf); eight of the dishes also included wine or brandy (six with white wine, one with red wine, and one with brandy); four contained flour or starch and one contained sugar.

### 3.1. General Composition of Meat Dishes

The proximate composition of the 12 analyzed dishes (Table 2) exhibited significant variability, with no clear influence of the type of meat, cooking technology used, or added ingredients.

The moisture content of the dishes ranged from 57.4% to 68.6%, except for “stewed veal—b” dish, which had a significantly higher moisture content (78.2%). This dish was prepared with a much higher quantity of vegetables than the others (110 g meat + 140 g vegetables), which explains the result. The Spanish Food Composition Database (BEDCA) [25] and the United States Department of Agriculture (USDA) FoodData Central [26] report similar moisture contents for various cooked beef (55.7–65.7%) and pork dishes (56.1–65.2%). The amount of protein present in meats varies among different muscles of pork and beef [27,28,29]. The culinary technique used could also affect the content of protein due to the higher or lower cooking loss that occurs [30,31,32]. All the analyzed dishes exhibited a high protein content, ranging from 24% to 34%, except for the “stewed veal—b” (18.2%) and “roasted pork meatballs” (16.6%). In the case of the “stewed veal—b”, the lower protein content was likely due to its higher moisture level. Conversely, the reduced protein content in the “roasted pork meatballs” was related with the dish’s higher fat content (15%). When comparing all the samples, no relevant differences were observed due to the type of meat or culinary method applied. According to BEDCA [25] and FoodData Central [26], the protein content of cooked beef ranges from 27.7% to 31%, while pork ranges from 27% to 28.8%. Similar findings have been reported in the literature [30,31,33].

In general, meat products contain little-to-no carbohydrates. However, other ingredients used in the dishes can contribute significantly to the carbohydrate content. The “roasted pork meatballs” exhibited a relatively high carbohydrate content (7.4%), primarily due to the sugar added in the sauce. Similarly, the “roasted pork tenderloin” contained 3% of carbohydrates, resulting from the use of corn starch in the recipe. However, although the recipes for “roasted veal round—b” and “stewed veal—b” included corn flour, only a negligible amount of carbohydrates was detected in these dishes.

Every recipe included salt as an ingredient, so it could be stated that sodium chloride is one of the most significant components of the ash fraction. During cooking, minerals and, in general, micronutrients can transfer from the meat to the sauces served in the dish [34]. The ash content varied between 0.9% and 2.3% for all the beef and pork dishes, with the grilled samples having the highest content.

Notable differences were found in the fat percentage of the dishes. Those with the highest fat content were “roasted pork meatballs” (15.1%), “grilled veal fillet—b” (13.1%), and “stewed veal—a” (9.9%), which correspond to the dishes with a lower moisture content. On the contrary, the dishes with the lowest fat content were “roasted veal round—a”, “stewed veal—b”, and “roasted pork tenderloin” (2.5–3.0%). None of these dishes showed results that differed significantly when compared to the available data [30,31,33]. The fat content in “grilled veal fillet—a” and “grilled veal fillet—b” was completely different (4.6 and 13.1%, respectively). Fat content can vary among different meat cuts with more or less fat content (leaner or fattier), the genetic, sex, breed, feeding system, or age [35]. According to FoodData Central [26], the differences in fat content of raw beef can vary between 2.4% in “eye of round” or “top of round” cuts and 20% in “ribeye” cuts.

Even more important than the total fat content of foods is the quality of this fat which is basically determined by their fatty acid profile. Table 3 shows the amount of the different fatty acid fractions supplied by each dish, and the corresponding ratios for all the analyzed samples. The complete fatty acid profiles of the 12 dishes are provided in the Supplementary Materials.

As is well known, SFAs in foods of animal origin are quite abundant. Dishes supplying the highest SFAs were the ones with the highest fat content, revealing that SFAs were the major fraction in meat fat (Table 3). Comparing the MUFA proportions in relation to the other two fractions (Supplementary Materials) with data reported by the literature for raw beef and pork cuts (beef: 40.9–51.5% of SFA, 25.4–49.9% of MUFA, and 2.9–10.7% of PUFA; pork: 36.9–41.7% of SFA, 26.9–46.8% of MUFA, and 14–32.5% of PUFA) [36], it is clear that the use of vegetable oils during cooking improved the fatty acid profile of the meat dishes compared to the raw meat. On the other hand, PUFA amounts were lower than SFAs and MUFAs, ranging between 0.11 and 1.45 g/100g in beef dishes, and between 0.48 and 1.77 g/100g in pork dishes, being always the omega-6 fatty acids that are most abundant. In both beef- and pork-based dishes, oleic acid served as the primary monounsaturated fatty acid, comprising 32–53% of the total fatty acid profile. Among saturated fatty acids, palmitic acid (18–26%) and stearic acid (7–16%) predominated. Regarding polyunsaturated fatty acids, linoleic acid, an omega-6 fatty acid, was the most prevalent, accounting for 4–18% of the profile (see Supplementary Materials).

A high omega-6/omega-3 ratio, and specifically, an insufficient omega-3 (EPA and DHA) consumption, are considered risk factors for cardiovascular and neurocognitive diseases [37,38]. As it has been pointed out before, the amount of omega-3 was very low in all dishes, and omega-6 predominated both in beef and pork. In consequence, all of them exhibited high omega-6/omega-3 ratios, ranging from 9.7 to 71.4 in beef dishes, and 31.6 to 56.7 in pork dishes. These ratios deviate significantly from 1:1/2:1, typically observed before the industrialization of society [39]. Molendi-Coste et al. [40], in their study on the composition and omega-6/omega-3 ratio of lunches provided by hospital and school catering services, also reported high ratios, highlighting the challenge of meeting omega-3 recommendations by using only natural sources of these fatty acids. In this context, all the meat-based dishes analyzed in this study exhibited low omega-3 fatty acid content (0.01–0.03g/100 g dish). Finally, the TFA amounts were always under 0.32g/100g dish, and in the case of pork dishes, under 0.1g/100g in nearly every sample.

It can be observed that in spite of the use of sunflower oil in “grilled veal fillet—b”, no significant differences were appreciated in the fatty acid profiles compared to “grilled veal fillet—a”.

As a consequence of the great differences found in the percentages of macronutrients among dishes, the energy values were also quite different, ranging from 96 kcal/100 g in “stewed veal—b” to 233 kcal/100 g in “roasted pork meatballs”.

All these results provide important information about the nutrients supplied by the different analyzed dishes. However, it is also crucial to understand how the digestion process affects nutrient digestibility and the oxidation status of the lipid fraction.

### 3.2. In Vitro Digestion Process Effects

The total digestibility and the protein and lipid digestibility (Table 4) were assessed after submitting the samples to an in vitro digestion process.

Three pork dishes showed total digestibility values (IVDMD) higher than 80%, whereas only two beef-based and one pork-based showed values lower than 70%. In vitro dry matter digestibility (IVDMD) indicates the extent to which foods or nutrients can be broken down and utilized by the body throughout the entire upper digestive process. A high dry matter digestibility value suggests efficient digestion and a potential valuable nutrient source. This information is critical for assessing the bioavailability of nutrients from different food sources, guiding dietary recommendations, and optimizing cooking techniques [41,42].

From a nutritional point of view, it is essential to consider protein digestibility [43], which determines the body’s ability to obtain essential amino acids [44,45,46]. The protein digestibility was higher than 90% in two pork dishes (“roasted pork loin” and “grilled pork loin”), but it was higher than 85% in nearly every sample. The influence of the animal species (pork or beef) in the protein digestibility of the dishes was not evident, as also noticed in the study carried out by Wen et al. [46], who did not find differences after intestinal digestion between pork, chicken, beef, and fish.

No significant effects of the cooking method used or added ingredients was observed. Lian et al. [47] did not find significant differences in protein digestibility (with values ranging from 83% to 85%) after the intestinal digestion of chicken wings cooked using different conventional methods. Similarly, Gawat et al. [48] did not observe any differences between lamb and goat samples cooked using microwave and sous vide methods. However, other studies have shown that cooking temperature can modulate the rate of protein digestion [5,43,49,50,51].

Additionally, some studies have highlighted the influence of other components, such as carbohydrates and fats, on protein digestibility. Lech and Reigh [52] reported that a high carbohydrate presence could decrease protein digestibility in fish. Ding et al. [53] found that an increase in fat content significantly improved the digestibility of pork protein (from 80% to 86%) and chicken protein (from 69% to 87%). Wakita et al. [54] observed that protein degradation after the in vitro gastric digestion of beef marinated with lemon juice was higher than in samples without marination, indicating that seasonings can affect meat digestion.

Lipid digestibility (IVLD) was measured as the concentration of the free fatty acids (FFAs) released from the meal into the luminal fluid and are available to be absorbed [55]. “Roasted pork loin—b” was the sample with the highest lipid digestibility (45.9%), which was consistent with the also-higher value observed in dry matter and protein digestibility. On the contrary, most of the pork samples showed values lower than 20%.

Zhou et al. [23] and Zhou et al. [56] found values of 35% and 8.73 for lipid digestibility in grilled beef burgers. West et al. [55] showed the fat bioaccessibility of 15.8% in beef Longissimus muscle cooked by Sous vide at 60 °C. They explained that a cooking temperature above 70 °C caused protein aggregation and meat toughness. Thus, a reduction in lipid digestibility occurred, as lipid is embedded as a droplet in the protein network.

### 3.3. Lipid Oxidation

Table 5 shows the lipid oxidation of the 12 dishes both before and after in vitro digestion, expressed as ppm MDA (mg MDA/kg of sample).

Grilled dishes (“grilled veal fillet—a”, “grilled veal fillet—b” and “grilled pork loin”) were the ones showing the lowest TBAR values before digestion (less than 0.85 ppm MDA). These dishes were cooked at a high temperature during a short time. Broncano et al. [57] and Domínguez et al. [58] also observed lower TBAR values in pig and foal products cooked by grilling compared with other technologies. These pointed out that lipid oxidation is more affected by cooking time than by temperature. On the contrary, three out of the four roasted dishes showed TBAR values higher than 4 ppm MDA. The use of vegetables or spices in the recipes seemed not to avoid the lipid oxidation that takes place during cooking.

During the in vitro digestion process, all dishes showed, in general, an increment in TBARs (31–609%). Antonini et al. [59] found significant increases in TBARs during in vitro digestion of beef burgers. Grilled and especially roasted samples, showed the greatest increments. “Roasted pork meatball” showed the highest lipid oxidation values (33 ppm MDA), being the dish with the highest increment during digestion (609%) but also with the highest values before cooking (4.65 ppm MDA). The minced process of meat that increases the surface exposure to air and thus to the oxygen, and its high fat content (15%) could explain the accumulation of secondary oxidation products derived from lipids. There are studies reporting a correlation between the amount of fat in meat and the amount of hydroperoxides, aldehydes, and TBARs after the digestion process [60,61,62]. On the contrary, stewed samples showed the lowest increases in lipid oxidation during digestion, and in the case of “stewed veal—a”, even a small decrease was observed. The TBARs of stewed dishes, both beef and pork, showed, in general, lower values after the digestion process compared with the rest of the samples. More data would be needed to conclude the potential positive effect of this type of cooking during the digestion process.

Kim and Hur [63] observed that oxidation during the in vitro digestion of pork patties could be significantly limited by the use of a potent antioxidant, such as dried tomato. Van Hecke et al. [64] found that the addition of herbs reduced MDA concentrations 2 to 3 times in cooked beef during in vitro digestion. However, in this work, the use of vegetables does not seem to avoid or limit the lipid oxidation process.

## 4. Conclusions

The general composition of the 12 dishes, cooked using different culinary methods, showed great variability. The protein content was not influenced by the type of meat or culinary technique used, with high values in all samples (more than 24%), except for “stewed veal—b” and “roasted pork meatballs.” The relatively high carbohydrate content in some dishes was attributed to the presence of other ingredients, such as sugar or corn flour. Significant differences were observed in the fat content of the dishes, with the highest percentage found in “roasted pork meatballs” and the lowest in “stewed veal—b.” The omega-6/omega-3 ratio was notably high in all samples, representing a risk factor for certain diseases. Regarding digestibility parameters, no clear influence of ingredients, cooking methods, or species was observed. “Roasted pork loin” exhibited the highest dry matter digestibility, as well as protein and lipid digestibility. Protein digestibility was high in all samples, with only “stewed pork cheeks” below 80%. Lipid digestibility showed greater variability. Concerning lipid oxidation, grilled dishes had the lowest TBAR values before digestion, whereas most roasted dishes exhibited TBAR values exceeding 4 ppm MDA. The inclusion of vegetables or spices in the recipes did not seem to prevent lipid oxidation during cooking. During in vitro digestion, all samples, except “stewed veal—a”, showed an increase in oxidation, with “roasted pork meatballs” presenting the greatest increase, likely due to its processing and high lipid content. These results contribute to a better understanding of the nutritional value of meat-based dishes prepared in catering services, considering also the effect of the digestion process.

## Figures and Tables

**Table 1:** General information about the 12 dishes categorized by meat type and cooking method.

Species	Dish	Cooking Method	Cooking Equipment	Temperature (°C)	Time (Min)	Ingredients
Beef	Grilled veal fillet—a	Grilling	Griddle	200	1	Extra-virgin olive oil and salt
	Grilled veal fillet—b	Grilling	Griddle	225	4	Sunflower oil, olive oil, salt, and garlic
	Roasted veal round—a	Roasting	Oven	1º: 75 2º: 250	120	Olive oil, salt, onion, carrot, and white wine
	Roasted veal round—b	Roasting	Oven	160	120	Extra-virgin olive oil, salt, onion, carrot, tomato, brandy, garlic, and corn flour
	Stewed veal—a	Stewing	Tilting pan	1º: 220 seal 2º: 180	105	Extra-virgin olive oil, salt, carrot, white wine, onion puree, peas, bay leaf, and garlic
	Stewed veal—b	Stewing	Vario Cook	110	60	Olive oil, salt, corn flour, tomato, pepper, onion, carrot, white wine, and garlic
Pork	Grilled pork loin	Grilling	Tilting pan	220-250	3	Extra-virgin olive oil and salt
	Roasted pork loin—a	Roasting	Oven	1º: 190 seal 2º: 200-220	35	Extra-virgin olive oil, salt, onion, corn starch, white wine, and golden apple
	Roasted pork loin—b	Roasting	Oven	180	90	Olive oil, salt, parsley, tomato, garlic, white wine, and onion
	Roasted pork meatballs	Roasting	Oven	165	60	Olive oil, salt, tomato, carrot, sugar, garlic, and onion
	Roasted pork tenderloin	Roasting	Oven	190	25	Olive oil, salt, white wine, onion, and corn starch
	Stewed pork cheeks	Stewing	Vario Cook	1º: 250 seal 2º: 150	120	Extra-virgin olive oil, salt, pepper, red wine, onion, leek, carrot, and garlic

**Table 2:** Composition of the 12 dishes categorized by meat type and cooking method.

Species	Cooking Method	Dish	Energy (kcal/100 g)	Moisture (%)	Protein (%)	Fat (%)	CH (%)	Ash (%)
Beef	Grilling	Grilled veal fillet—a	167	66.0 ± 0.5 de	31.4 ± 1.5 def	4.6 ± 0.2 b	0.0	1.5 ± 0.0 e
		Grilled veal fillet—b	230	57.4 ± 0.6 a	26.2 ± 2.1 bcd	13.1 ± 0.7 e	1.8	1.3 ± 0.0 de
	Roasting	Roasted veal round—a	160	67.3 ± 0.1 efg	34.0 ± 0.5 f	2.6 ± 0.2 a	0.0	1.1 ± 0.1 bc
		Roasted veal round—b	167	62.9 ± 0.3 bc	31.3 ± 1.5 def	4.5 ± 0.4 b	0.2	1.1 ± 0.1 b
	Stewing	Stewed veal—a	211	61.4 ± 0.5 b	30.5 ± 1.5 cdef	9.9 ± 0.6 d	0.0	1.2 ± 0.1 bcd
		Stewed veal—b	96	78.2 ± 1.4 h	18.2 ± 1.2 a	2.5 ± 0.2 a	0.1	0.9 ± 0.0 a
Pork	Grilling	Grilled pork loin	172	63.9 ± 1.1 cd	25.3 ± 1.1 bc	7.4 ± 0.3 c	1.0	2.3 ± 0.1 g
	Roasting	Roasted pork loin—a	166	68.2 ± 0.2 fg	32.4 ± 1.7 ef	3.9 ± 0.3 b	0.0	1.5 ± 0.0 e
		Roasted pork loin—b	177	66.3 ± 1.6 ef	27.6 ± 2.0 bcde	7.4 ± 0.3 c	0.0	1.2 ± 0.1 bcd
		Roasted pork meatballs	233	59.0 ± 0.5 a	16.6 ± 0.2 a	15.1 ± 0.6 f	7.7	1.6 ± 0.0 f
		Roasted pork tenderloin	136	68.6 ± 0.9 g	24.1 ± 0.7 b	3.0 ± 0.3 a	3.0	1.3 ± 0.0 d
	Stewing	Stewed pork cheeks	207	62.6 ± 0.3 bc	33.8 ± 2.2 f	8.0 ± 0.4 c	0.0	1.1 ± 0.0 b

Carbohydrates (CH) were obtained by difference. The energy values were calculated as 4 Kcal per gram of protein and carbohydrates, and 9 Kcal per gram of fat. Moisture, protein, fat, and ash values represent the means and standard deviation of three replicates. An ANOVA and the Tukey post hoc test were applied to analyze the differences among the dishes. For each parameter, different letters indicate significant differences among samples (*p* < 0.05).

**Table 3:** Fatty acid amount (g/100 g Dish) and some relevant ratios of the 12 dishes categorized by the type of meat and cooking method.

Species	Cooking Method	Dish	SFA g/100 g Dish	MUFA g/100 g Dish	PUFA g/100 g Dish	ω3 g/100 g Dish	ω6 g/100 g Dish	ω6/ω3	PUFA/SFA	UFA/SFA	TFA g/100 g Dish
**Beef**	Grilled	Grilled veal fillet—a	1.05 ± 0.00 ab	2.08 ± 0.00 d	0.47 ± 0.00 cd	0.01 ± 0.00 a	0.46 ± 0.00 c	71.4 ± 3.1	0.4 ± 0.0	2.4 ± 0.0	0.11 ± 0.00 d
		Grilled veal fillet—b	3.12 ± 0.29 f	5.40 ± 0.23 i	1.45 ± 0.06 g	0.02 ± 0.00 ef	1.43 ± 0.06 f	66.6 ± 9.2	0.5 ± 0.1	2.2 ± 0.3	0.24 ± 0.00 g
	Roasted	Roasted veal round—a	0.92 ± 0.01 a	0.82 ± 0.03 a	0.30 ± 0.00 b	0.01 ± 0.00 a	0.29 ± 0.00 b	42.0 ± 7.1	0.3 ± 0.0	1.2 ± 0.0	0.15 ± 0.01 e
		Roasted veal round—b	1.77 ± 0.01 c	1.61 ± 0.01 c	0.20 ± 0.00 a	0.02 ± 0.00 f	0.17 ± 0.00 a	7.2 ± 0.1	0.1 ± 0.0	1.0 ± 0.0	0.10 ± 0.01 d
	Stewed	Stewed veal—a	2.68 ± 0.00 e	4.28 ± 0.02 g	0.52 ± 0.00 cd	0.02 ± 0.00 de	0.50 ± 0.00 c	27.6 ± 0.6	0.2 ± 0.0	1.8 ± 0.0	0.20 ± 0.02 f
		Stewed veal—b	0.98 ± 0.04 ab	0.95 ± 0.03 ab	0.11 ± 0.01 a	0.01 ± 0.00 ab	0.10 ± 0.00 a	9.7 ± 1.0	0.1 ± 0.0	1.1 ± 0.1	0.05 ± 0.01 b
**Pork**	Grilled	Grilled pork loin	2.21 ± 0.01 d	3.22 ± 0.03 f	0.71 ± 0.02 ef	0.02 ± 0.00 de	0.69 ± 0.02 de	36.8 ± 3.3	0.3 ± 0.0	1.8 ± 0.0	0.09 ± 0.01 cd
	Roasted	Roasted pork loin—a	1.31 ± 0.00 b	1.2 ± 0.00 b	0.60 ± 0.00 de	0.02 ± 0.00 de	0.58 ± 0.00 cd	31.6 ± 0.6	0.5 ± 0.0	1.4 ± 0.0	0.05 ± 0.00 ab
		Roasted pork loin—b	2.58 ± 0.08 e	2.68 ± 0.04 e	0.64 ± 0.09 ef	0.01 ± 0.00 bc	0.62 ± 0.09 de	46.6 ± 11.2	0.2 ± 0.0	1.3 ± 0.1	0.07 ± 0.00 bc
		Roasted pork meatballs	5.13 ± 0.24 g	4.76 ± 0.19 h	1.77 ± 0.06 h	0.03 ± 0.00 g	1.74 ± 0.06 g	56.7 ± 1.3	0.3 ± 0.0	1.3 ± 0.1	0.17 ± 0.01 e
		Roasted pork tenderloin	0.96 ± 0.00 ab	1.00 ± 0.00 ab	0.48 ± 0.00 c	0.01 ± 0.00 a	0.47 ± 0.00 c	65.4 ± 1.9	0.5 ± 0.0	1.5 ± 0.0	0.03 ± 0.00 a
	Stewed	Stewed pork cheeks	2.20 ± 0.02 d	3.35 ± 0.04 f	0.72 ± 0.02 f	0.02 ± 0.00 cd	0.70 ± 0.02 e	44.3 ± 3.5	0.3 ± 0.0	1.8 ± 0.0	0.07 ± 0.00 bc

SFA: saturated fatty acid; MUFA: monounsaturated fatty acid; PUFA: polyunsaturated fatty acid; TFA: trans fatty acid; UFA: unsaturated fatty acid. The values represent the means and standard deviations of two replicates. An ANOVA and the Tukey post hoc test were applied to analyze the differences among the dishes. For each parameter, different letters indicate significant differences among the samples (*p* < 0.05).

**Table 4:** In vitro digestibility of the 12 dishes categorized by meat type and cooking method.

Species	Cooking Method	Dish	IVDMD (%)	IVPD (%)	IVLD (%)
Beef	Grilling	Grilled veal fillet—a	69.7 ± 1.5 a	86.0 ± 0.7 bcd	40.5 ± 2.1 d
		Grilled veal fillet—b	73.5 ± 4.1 abc	80.4 ± 2.3 ab	20.1 ± 1.5 bc
	Roasting	Roasted veal round—a	74.1 ± 2.2 abc	87.0 ± 2.1 cde	32.0 ± 1.4 cd
		Roasted veal round—b	73.6 ± 5.2 abc	81.8 ± 8.4 abc	7.3 ± 1.0 a
	Stewing	Stewed veal—a	73.0 ± 3.3 abc	87.4 ± 1.4 cde	37.6 ± 0.8 d
		Stewed veal—b	68.9 ± 6.0 a	85.3 ± 3.7 bcd	32.2 ± 14.2 cd
Pork	Grilling	Grilled pork loin	81.3 ± 1.6 bcd	90.3 ± 1.6 de	11.6 ± 2.5 ab
	Roasting	Roasted pork loin—a	71.1 ± 1.9 ab	85.7 ± 1.0 bcd	26.0 ± 2.0 c
		Roasted pork loin—b	86.2 ± 3.2 d	93.0 ± 1.5 e	45.9 ± 1.9 d
		Roasted pork meatballs	82.8 ± 6.0 cd	85.7 ± 2.8 bcd	14.2 ± 1.0 ab
		Roasted pork tenderloin	77.2 ± 2.6 abcd	87.9 ± 0.4 cde	20.7 ± 4.2 bc
	Stewing	Stewed pork cheeks	68.5 ± 3.7 a	77.1 ± 2.8 a	8.1 ± 1.9 a

IVDMD: in vitro dry matter digestibility; IVPD: in vitro protein digestibility; and IVLD: in vitro lipid digestibility. The values represent the means and standard deviation of three replicates. An ANOVA and the Tukey post hoc test were applied to analyze differences among the dishes. For each parameter, different letters indicate significant differences among samples (*p* < 0.05).

**Table 5:** Lipid oxidation (mg MDA/kg sample) of the 12 dishes (categorized by meat type and cooking method) before and after in vitro digestion.

Species	Cooking Method	Dish	Cooked (mg MDA/kg Sample)	Digested (mg MDA/kg Sample)	*p*-Value	Oxidation Increase (%)
Beef	Grilling	Grilled veal fillet—a	0.40 ± 0.02 a	1.53 ± 0.06 a	***	282
		Grilled veal fillet—b	0.83 ± 0.04 a	4.54 ± 0.31 bc	***	447
	Roasting	Roasted veal round—a	2.09 ± 0.04 c	5.75 ± 0.26 c	***	175
		Roasted veal round—b	4.23 ± 0.14 f	7.51 ± 0.50 d	***	77
	Stewing	Stewed veal—a	3.04 ± 0.25 de	1.89 ± 0.08 a	**	−38
		Stewed veal—b	1.33 ± 0.11 b	2.10 ± 0.11 a	*	58
Pork	Grilling	Grilled pork loin	0.75 ± 0.02 a	1.27 ± 0.12 a	**	69
	Roasting	Roasted pork loin—a	4.61 ± 0.23 f	15.42 ± 0.46 f	***	234
		Roasted pork loin—b	2.61 ± 0.03 d	9.95 ± 0.27 e	***	281
		Roasted pork meatballs	4.65 ± 0.21 f	33.01 ± 1.56 g	***	610
		Roasted pork tenderloin	2.94 ± 0.18 de	10.71 ± 0.42 e	***	264
	Stewing	Stewed pork cheeks	3.11 ± 0.13 e	4.08 ± 0.35 b	*	31

The values represent the mean and standard deviation of three replicates. An ANOVA and the Tukey post hoc test were applied to analyze the differences among the dishes. For each parameter, different letters indicate significant differences (*p* < 0.05). The Student *t*-test was carried out to assess the differences between samples before and after digestion for each dish. p: significance. *: *p* < 0.05; **: *p* < 0.01; ***: *p* < 0.001.

## Data Availability

The original contributions presented in the study are included in the article/Supplementary Materials, further inquiries can be directed to the corresponding author.

## Supplementary Materials

The following supporting information can be downloaded at: https://www.mdpi.com/article/10.3390/foods14050789/s1, Table S1: Fatty acid composition (g/100 g total fatty acids) of the 12 dishes categorized by type of meat and cooking method.
