# Supplementary material for: Beef- and Pork-Based Dishes from Catering Services: Composition and In Vitro Digestion Effects on Digestibility and Lipid Oxidation
Source: Foods. 2025 Feb 25;14(5):789. doi: 10.3390/foods14050789 (PMC11899560; doi:10.3390/foods14050789)
Supplement: Supplementary file 1 [file foods-14-00789-s001.zip › foods-3470576-supplementary.pdf]

Table S1. Fatty acid composition (g/100 g total fatty acids) of the 12 dishes categorized by type of meat and cooking method.

**Species**	**Beef**						**Pork**					
**Cooking method**	**Grilling**		**Roasting**		**Stewing**		**Grilling**	**Roasting**				**Stewing**
**Dish**	Grilled veal fillet - a	Grilled veal fillet - b	Roasted veal round - a	Roasted veal round - b	Stewed veal - a	Stewed veal - b	Grilled pork loin	Roasted pork loin - a	Roasted pork loin - b	Roasted pork meatballs	Rorted pork tenderloin	Stewed pork cheeks
Caprylic acid C8:0	0.05 ± 0	0.06 ± 0	0.1 ± 0.04	0.05 ± 0	0.03 ± 0.01	0.06 ± 0.03	0.06 ± 0.01	0.06 ± 0	0.06 ± 0	0.03 ± 0	0.04 ± 0.01	0.06 ± 0
Capric acid C10:0	0.02 ± 0.01	0.06 ± 0	0.07 ± 0.05	0.03 ± 0	0.03 ± 0	0.04 ± 0.01	0.04 ± 0	0.07 ± 0.02	0.06 ± 0	0.04 ± 0	0.04 ± 0	0.05 ± 0.01
Lauric acid C12:0	0.03 ± 0	0.04 ± 0	0.05 ± 0.01	0.04 ± 0	0.05 ± 0	0.05 ± 0	0.05 ± 0	0.06 ± 0	0.08 ± 0	0.1 ± 0	0.06 ± 0	0.04 ± 0
Myristic acid C14:0	0.72 ± 0.04	0.86 ± 0.04	1.57 ± 0.11	2.32 ± 0.02	1.55 ± 0.01	2.14 ± 0.07	0.9 ± 0.02	1.02 ± 0	1.18 ± 0.02	1.3 ± 0.04	0.96 ± 0.02	0.72 ± 0.02
Palmitic acid C16:0	18.42 ± 0.11	18.85 ± 1.18	22.28 ± 0.18	24.96 ± 0.27	20.69 ± 0.03	24.7 ± 0.88	21.63 ± 0.11	23.73 ± 0.03	24.18 ± 0.79	25.64 ± 2.7	23.25 ± 0.05	20.99 ± 0.15
t-Palmitoleic acid C16:1	0.11 ± 0.01	0.13 ± 0	0.33 ± 0.08	0.5 ± 0.01	0.2 ± 0.01	0.39 ± 0.01	0.31 ± 0	0 ± 0	0.29 ± 0	0.41 ± 0.01	0.39 ± 0.04	0.24 ± 0.01
Palmitoleic acid C16:0	1.56 ± 0.11	1.56 ± 0.05	2.52 ± 0.33	5.17 ± 0.67	3.04 ± 0.03	3.71 ± 0.14	2.32 ± 0.03	1.71 ± 0.02	2.05 ± 0.03	1.92 ± 0.05	1.68 ± 0.04	2.28 ± 0.05
Stearic acid C18:0	7.03 ± 0.04	7.57 ± 1.27	15.27 ± 0.13	15.34 ± 0.17	9.24 ± 0.07	15.94 ± 1.14	10.17 ± 0.16	14.51 ± 0.03	15.33 ± 1.03	14.12 ± 0.73	12.54 ± 0.03	10.24 ± 0.15
Elaidic C18:1	1.45 ± 0.02	1.56 ± 0.02	5.09 ± 0.15	1.2 ± 0.13	1.85 ± 0.27	1.01 ± 0.12	0.36 ± 0.05	0.12 ± 0	0.32 ± 0.05	0.39 ± 0.05	0.13 ± 0.01	0.23 ± 0.06
Oleic acid 18:1	53.08 ± 0.07	49.63 ± 1.9	32 ± 1.59	40.22 ± 0.42	52.7 ± 0.23	41.98 ± 1.26	46.65 ± 0.6	34.73 ± 0.02	41.04 ± 0.63	36.62 ± 1.28	34.34 ± 0.15	47.6 ± 0.6
Vaccenic acid C18:1	2.59 ± 0.01	2.48 ± 0.08	2.16 ± 0.21	1.73 ± 0.01	2.33 ± 0.01	1.81 ± 0.17	3.62 ± 0.03	0 ± 0	2.95 ± 0.03	2.6 ± 0.3	2.99 ± 0.01	3.63 ± 0.21
t-Linoleic acid C18:2	0.03 ± 0	0.04 ± 0	0.09 ± 0.02	0.21 ± 0.01	0.11 ± 0	0.13 ± 0.01	0.02 ± 0	0.15 ± 0.03	0.01 ± 0.01	0.02 ± 0	0.01 ± 0	0.04 ± 0.03
c-t Linoleic acid C18:2	0.27 ± 0.01	0.46 ± 0.03	0.94 ± 0.54	0.65 ± 0.05	0.36 ± 0.02	0.73 ± 0.17	0.46 ± 0.1	0.99 ± 0.18	0.36 ± 0.01	0.33 ± 0.02	0.36 ± 0.03	0.39 ± 0.04
t-c Linoleic acid C18:2	0.07 ± 0	0.11 ± 0.01	0.24 ± 0.1	0.13 ± 0	0.09 ± 0	0.16 ± 0.04	0.11 ± 0.02	0.2 ± 0.01	0.09 ± 0.01	0.09 ± 0	0.08 ± 0.01	0.1 ± 0
Linoleic acid C18:2	11.79 ± 0.04	13.43 ± 0.5	12.61 ± 0.19	4.33 ± 0.04	5.93 ± 0.03	4.35 ± 0.15	9.93 ± 0.13	17.96 ± 0.13	8.35 ± 0.14	13.16 ± 0.39	18.12 ± 0.06	9.81 ± 0.25
Arachidic acid C20:0	0.39 ± 0.03	0.42 ± 0.06	0.18 ± 0.1	0.16 ± 0	0.24 ± 0	0.18 ± 0.01	0.28 ± 0	0.26 ± 0	0.27 ± 0.02	0.23 ± 0.01	0.2 ± 0	0.28 ± 0.01
γ-Linolenic acid C18:3	0.19 ± 0.01	0.16 ± 0.01	0.2 ± 0.06	0.13 ± 0.01	0.21 ± 0	0.16 ± 0.05	0.75 ± 0.02	0 ± 0	0.68 ± 0.01	0.94 ± 0.1	0.64 ± 0.06	0.72 ± 0.01
Eicosenoic acid C20:1	0.35 ± 0.02	0.3 ± 0.01	0.25 ± 0.01	0.47 ± 0.03	0.24 ± 0.01	0.4 ± 0.01	0.33 ± 0.02	0.49 ± 0.02	0.34 ± 0.01	0.42 ± 0.07	0.55 ± 0.06	0.31 ± 0.01
α -Linolenic acid C18:3	0.11 ± 0	0.13 ± 0	0.28 ± 0.06	0.49 ± 0.01	0.21 ± 0	0.29 ± 0.03	0.12 ± 0.01	0.25 ± 0	0.08 ± 0.02	0.11 ± 0	0.09 ± 0.01	0.09 ± 0.01
Eicosadienoic acid C20:2	0.03 ± 0.01	0.18 ± 0	0.11 ± 0.06	0.07 ± 0.01	0.02 ± 0	0.07 ± 0.02	0.21 ± 0.15	0.1 ± 0	0.23 ± 0.13	0.63 ± 0.04	0.11 ± 0	0.25 ± 0.07
Behenic acid C22:0	0.14 ± 0.01	0.12 ± 0.02	0.05 ± 0.06	0 ± 0.01	0.01 ± 0	0.01 ± 0.01	0.02 ± 0.02	0 ± 0	0.03 ± 0.01	0 ± 0	0.07 ± 0	0.07 ± 0.01
Brassidic acid C22:1	1.08 ± 0.05	0.07 ± 0.01	0 ± 0	0.09 ± 0.01	0.05 ± 0.01	0.1 ± 0.02	0.14 ± 0.01	0.26 ± 0.01	0.12 ± 0.01	0.17 ± 0.01	0.17 ± 0.01	0.11 ± 0.03
Erucic acid C22:1	0 ± 0	1.02 ± 0.04	2.66 ± 0.13	1.17 ± 0.04	0.3 ± 0	0.9 ± 0.02	0.83 ± 0.07	2.11 ± 0	0.44 ± 0.01	0.55 ± 0.02	2.28 ± 0.04	1.2 ± 0.02
Eicosatrienoic acid C20:3	0 ± 0	0 ± 0	0 ± 0	0 ± 0	0 ± 0	0 ± 0	0 ± 0	0 ± 0	0 ± 0	0 ± 0	0 ± 0	0 ± 0
Arachidonic acid C20:4	0.48 ± 0	0.48 ± 0.02	0.49 ± 0.22	0.2 ± 0.02	0.39 ± 0	0.29 ± 0.06	0.27 ± 0.04	0.41 ± 0	1.17 ± 1.44	0 ± 0	0.25 ± 0.01	0.32 ± 0.01
Eicosapentaenoic acid C20:5	0.07 ± 0.01	0.08 ± 0.02	0.05 ± 0.01	0.02 ± 0	0.03 ± 0	0.03 ± 0.01	0.03 ± 0	0.02 ± 0	0.01 ± 0	0.02 ± 0	0.02 ± 0	0.03 ± 0
Nervonic acid C24:1	0 ± 0	0.18 ± 0	0.44 ± 0.28	0.17 ± 0	0.11 ± 0	0.22 ± 0.02	0.21 ± 0.02	0.47 ± 0	0.14 ± 0.01	0.04 ± 0	0.44 ± 0.04	0.07 ± 0.05
Docosahexaenoic C22:6	0 ± 0	0 ± 0	0 ± 0	0.15 ± 0.02	0 ± 0	0.19 ± 0.05	0.16 ± 0.03	0.31 ± 0	0.14 ± 0	0.14 ± 0.01	0.17 ± 0	0.13 ± 0.02
SFA	28.3 ± 0.1	30.5 ± 2.8	42.1 ± 0.6	48.1 ± 0.2	34.9 ± 0.0	46.8 ± 1.8	35.5 ± 0.1	41.4 ± 0.0	43.2 ± 1.4	43.4± 2.1	38.8 ± 0.1	34.7 ± 0.3
MUFA	56.0 ± 0.1	52.9 ± 2.3	37.5 ± 1.2	43.8 ± 0.4	55.7 ± 0.3	45.3 ± 1.4	51.6 ± 0.5	37.8 ± 0.0	44.9 ± 0.7	40.2 ± 1.6	40.6 ± 0.1	52.8 ± 0.7
PUFA	12.7 ± 0.0	14.2 ± 0.6	13.7 ± 0.2	5.4 ± 0.1	6.8 ± 0.0	5.4 ± 0.3	11.5 ± 0.3	19.0 ± 0.1	10.7 ± 1.4	15.0 ± 0.5	19.4 ± 0.1	11.4 ± 0.3
ω3	0.2 ± 0.00	0.2 ± 0.0	0.3 ± 0.1	0.7 ± 0.0	0.2 ± 0.0	0.5 ± 0.1	0.3 ± 0.0	0.6 ± 0.0	0.2 ± 0.0	0.3 ± 0.0	0.3 ± 0.0	0.3 ± 0.0
ω6	12.5 ± 0.0	14.0 ± 0.6	13.4 ± 0.2	4.7 ± 0.1	6.6 ± 0.0	4.9 ± 0.2	11.2 ± 0.3	18.5 ± 0.1	10.4 ± 1.5	14.7 ± 0.5	19.1 ± 0.1	11.1 ± 0.3
ω6/ω3	71.4 ± 3.1	66.6 ± 9.2	42.0 ± 7.1	7.2 ± 0.1	27.6 ± 0.6	9.7 ± 1.0	36.8 ± 3.3	31.6 ± 0.6	46.6 ± 11.2	56.7 ± 1.3	65.4 ± 1.9	44.3 ± 3.5
PUFA/SFA	0.4 ± 0.00	0.5 ± 0.1	0.3 ± 0.0	0.1 ± 0.0	0.2 ± 0.0	0.1 ± 0.0	0.3± 0.0	0.5 ± 0.0	0.2 ± 0.0	0.3 ± 0.0	0.5 ± 0.0	0.3 ± 0.0
UFA/SFA	2.4 ± 0.00	2.2 ± 0.3	1.2 ± 0.0	1.0 ± 0.0	1.8 ± 0.0	1.1 ± 0.1	1.8 ± 0.0	1.4 ± 0.0	1.3 ± 0.1	1.3 ± 0.1	1.5 ± 0.0	1.8 ± 0.0
TFA	3.00 ± 0.0	2.4 ± 0.0	6.7 ± 0.6	2.8 ± 0.2	2.7 ± 0.3	2.5 ± 0.3	1.4 ± 0.1	1.7 ± 0.2	1.2 ± 0.0	1.4 ± 0.0	1.2 ± 0.1	1.1 ± 0.1

SFA: saturated fatty acids; MUFA: monounsaturated fatty acids; PUFA: polyunsaturated fatty acids; UFA: unsaturated fatty acids; TFA: trans fatty acids.
